# Microarray-based cancer prediction using single genes

**DOI:** 10.1186/1471-2105-12-391

**Published:** 2011-10-07

**Authors:** Xiaosheng Wang, Richard Simon

**Affiliations:** 1 Biometric Research Branch, National Cancer Institute, National Institutes of Health, Rockville, MD 20852, USA

## Abstract

**Background:**

Although numerous methods of using microarray data analysis for cancer classification have been proposed, most utilize many genes to achieve accurate classification. This can hamper interpretability of the models and ease of translation to other assay platforms. We explored the use of single genes to construct classification models. We first identified the genes with the most powerful univariate class discrimination ability and then constructed simple classification rules for class prediction using the single genes.

**Results:**

We applied our model development algorithm to eleven cancer gene expression datasets and compared classification accuracy to that for standard methods including Diagonal Linear Discriminant Analysis, *k*-Nearest Neighbor, Support Vector Machine and Random Forest. The single gene classifiers provided classification accuracy comparable to or better than those obtained by existing methods in most cases. We analyzed the factors that determined when simple single gene classification is effective and when more complex modeling is warranted.

**Conclusions:**

For most of the datasets examined, the single-gene classification methods appear to work as well as more standard methods, suggesting that simple models could perform well in microarray-based cancer prediction.

## Background

Recent advances in microarray technology have made it feasible to rapidly measure the expression levels of tens of thousands of genes in a single experiment at a reasonable expense [1]. This technology has facilitated the molecular exploration of cancer [2-9]. For medical applications, gene expression profiling can be used to develop classifiers of prognosis or sensitivity to particular treatments. A large literature on the development and validation of predictive classifiers has emerged [10]. Most of the classifiers developed have involved complex models containing numerous genes [5,11-16]. This has limited the interpretability of the classifiers and lack of interpretability hampers the acceptance of such diagnostic tools. Classification models based on numerous genes can also be more difficult to transfer to other assay platforms which may be more suitable for clinical application. Several authors have suggested that simple models could perform well in some cases of microarray-based cancer prediction [17-23].

The development of a molecular classifier includes gene selection and classification rule generation. A variety of gene selection strategies have been used. These include univariate gene selection and more complex multivariate methods. In [3], [17] and [24], the authors investigated classification based on a small number of selected gene pairs. In [20], the authors explored the use of one or two genes to perform tumor classifications. These investigations indicated that for the data examined, classifiers could be developed containing few genes that provided classification accuracy comparable to that achieved by more complex models. Some more complex algorithms have been used to select few genes for classification, but often overfit the data [15,25-29].

Many different classification rules have been proposed for high dimensional predictive classification including Support Vector Machines (SVM), Diagonal Linear Discriminant Analysis (DLDA), Artificial Neural Network (ANN), Prediction Analysis of Microarrays (PAM), Naïve Bayes (NB), *k*-Nearest Neighbor (*k*-NN), Nearest Centroid (NC), Decision Tree (DT), Random Forest (RF), Rough Set (RS) [30], Emerging Pattern (EP) [31] etc. Most of these methods produce "black-box" models, in which class predication is based on mathematical formulae which are difficult to interpret.

In this study, we explored the usefulness of very simple single gene classification models for molecular classification of cancer. Although in [20], the authors have investigated the use of single genes for classification of cancer, the applicability of that method was limited in that the authors identified multiple single genes potentially having good classification performance instead of determining one which would be validated and used for cancer prediction.

We compared the performance of the single gene models to that of a wide variety of more standard models using eleven publicly available gene expression datasets (http://linus.nci.nih.gov/~brb/DataArchive_New.html) [32]. We also compared the performance of single gene classifiers to a wide range of standard classifiers on the datasets evaluated in [33].

## Results

Table 1 lists the LOOCV results for single gene classification using the t-test and the WMW test for gene selection. For comparison, LOOCV results obtained by using the DLDA, *k*-NN, SVM and RF methods are also listed in Table 1. The classification results based on split-sample rather than LOOCV evaluation are presented in Table S1 and Table S2 (Additional file 1).

**Table 1:** The LOOCV classification accuracy (%)

Method Dataset	SGC-t	SGC-W	DLDA	*k*-NN	SVM	RF
Melanoma	97*	96**	97*	97*	97*	97*

Breast Cancer 1	63**	69*	61	53	52	43

Brain Cancer	80*	77**	65	73	60	70

Breast Cancer 2	58	50	73*	67**	73*	67**

Gastric Tumor	89	80	81	96**	97*	95

Lung Cancer 1	98*	95**	95**	98*	98*	98*

Lung Cancer 2	93**	93**	99*	99*	99*	99*

Lymphoma	74*	71**	66	52	59	57

Myeloma	68	67	75	78**	74	79*

Pancreatic Cancer	69**	90*	63	61	65	55

Prostate Cancer	89**	89**	78	93*	93*	93*

Note:

1 SGC-t: Single Gene Classifier with the t-test gene selection method.

2 SGC-W: Single Gene Classifier with the WMW gene selection method.

3 for each dataset, the highest classification accuracy is highlighted with a single asterisk and the second highest is highlighted with a double asterisk.

From Table 1, we can see that the Melanoma is an easily-classified dataset for which all the methods exhibit very high classification accuracy. In contrast, Breast Cancer 1 is a difficult dataset for which the standard methods show relatively low classification accuracy, whereas the single gene classifiers based on WMW and t-test show the best and second best results, respectively. In the Brain Cancer dataset, the t-test and the WMW single gene classifiers achieved the highest and second highest classification accuracy, respectively. In the Breast Cancer 2 dataset, the WMW and the t-test single gene classifiers show poorer accuracy than other methods. In the Gastric Tumor dataset, the WMW and the t-test classifiers show poorer accuracy than the k-NN, SVM and RF classifiers but are comparable to the DLDA classifier. In the Lung Cancer 1, Lung Cancer 2, Myeloma and Prostate Cancer datasets, the classification results obtained by the WMW and the t-test single gene classifiers are close to those obtained by the other four methods. Surprisingly, in the Lymphoma and the Pancreatic Cancer datasets, the classification results obtained by the WMW and the t-test single gene classifiers are much better than those obtained by the other four methods. For the evaluations based on separating each dataset into training and test sets, we obtained similar conclusions (see Table S1 and Table S2 of Additional file 1).

The number of genes used for building the classifiers averaged across the loops of the cross validation is listed in Table 2 for each method. From this table, we can see that the DLDA, *k*-NN, SVM and RF have used a large number of genes for constructing the classifiers in most of the eleven datasets. The number of genes in the classifiers constructed in the cases of separating samples into one training set and one test set is presented in Table S3 and Table S4 (Additional file 1).

**Table 2:** The mean number of genes in classifiers

Method Dataset	SGC-t	SGC-W	DLDA	*k*-NN	SVM	RF
Melanoma	1	1	7200	7200	7200	7200

Breast Cancer 1	1	1	17	17	17	15

Brain Cancer	1	1	14	14	14	14

Breast Cancer 2	1	1	176	176	176	176

Gastric Tumor	1	1	848	848	848	848

Lung Cancer 1	1	1	7472	7472	7472	7472

Lung Cancer 2	1	1	3207	3207	3207	3207

Lymphoma	1	1	2	2	2	2

Myeloma	1	1	169	169	169	169

Pancreatic Cancer	1	1	56	56	56	44

Prostate Cancer	1	1	798	798	798	798

Generally speaking, in the datasets with small sample sizes such as those for Melanoma, Brain Cancer, Lung Cancer 1, Lymphoma and Pancreatic Cancer, the single gene classifiers showed better or comparable classification results compared with the standard methods. In the datasets with relatively large sample sizes like the Lung Cancer 2, Gastric Tumor and Myeloma, the single gene classifiers showed poorer results. One possible explanation is that complex models require larger datasets for training and in some cases may be overfit for smaller datasets. The comparative results were not very related to the number of genes in the dataset. All datasets included many thousands of genes and as noted in [34], a good classifier from high-dimensional microarray data can involve a short gene list if there are many genes with large differences in expression between the classes. Clearly, in the Melanoma, Gastric Tumor, Lung Cancer 1, Lung Cancer 2 and Prostate Cancer dataset, there are many genes with large differences in expression between the classes so that it is not difficult to find a single gene on which to base a good classifier. In such cases, it is unnecessary for the standard algorithms to use so many genes in constructing classifiers for these datasets (see Table 2). Actually, the single gene classifiers achieve near-optimal classification results in these datasets. In contrast, if there are very few genes with large differences in expression between the classes, it will be difficult to build an effective single gene classifier because the gene selected may be the noise-gene with the greatest apparent degree of differential expression. In some cases, however, it might be equally difficult for complex algorithms to produce good classifiers for this sort of dataset, particularly when the sample size is small and overfitting is likely to occur. This might explain why the single gene classifiers performed better than complex classifiers in some difficult small sample datasets like the Brain Cancer, Lymphoma and Pancreatic Cancer.

Single gene classifiers are more influenced by selection of noise genes than standard methods. Some ''noise'' genes could have good t-test or WMW test statistics in the training set, and if such genes were selected for building the single gene classifiers, the performance of the classifiers would be poorer than the classifiers built based on a longer gene list. In the Breast Cancer 2 and Myeloma datasets, it was likely that the selection of ''noise'' genes had contributed to the poor results of the single gene classifiers. In fact, in the Breast Cancer 2 dataset, we found one gene in the list of five genes with the smallest t-test p-value, which could result in 73% classification accuracy, and in the Myeloma dataset, we found one gene in the list of 10 genes with the smallest t-test p-value, which could result in 92% classification accuracy. Both results are much better than those obtained by using the present gene selection methods (see Table 1). Therefore, sometimes it might be better to include a longer gene list in classifiers to prevent from falling into the trap of noise genes.

Table 3 explores how the performance of the single gene classifiers and the standard classifiers varies with some characteristics of the datasets. We calculated the smallest univariate t-test p-value and the corresponding t-statistic, the largest mean gene expression fold change between the classes, the total number of genes significantly different between the classes at 0.001 significance level, the average classification accuracy of the standard classifiers and the average classification accuracy of the single gene classifiers for each dataset. From this table, we can see that for the Melanoma, Gastric Tumor, Lung Cancer 1, Lung Cancer 2 and Prostate Cancer datasets, a large number of statistically significant genes were identified and used for building the classifiers by the standard algorithms, while for the Breast Cancer 1, Brain Cancer, Lymphoma and Pancreatic Cancer datasets, the number of statistically significant genes was quite limited so that the standard algorithms performed much more poorly on these datasets and the single gene classifiers had consistently improved classification performance compared to the standard classifiers in these datasets. This table also indicates that the fold change is highly related to the classification accuracy. In the Melanoma, Lung Cancer 1 and Lung Cancer 2 dataset, the fold changes are huge and the classification accuracies are extremely high. In the Gastric Tumor and Prostate Cancer datasets, although the numbers of statistically significant genes are also large, the fold changes are not as notable as those in the aforementioned three datasets, and therefore the classification accuracies in the latter are inferior to those in the three former datasets. Fold change affects the classification accuracy more than the number of significant genes. One evident example is that the significant gene number in the Brain Cancer dataset is less than those in the Breast Cancer 1 and Pancreatic Cancer dataset, whereas the classification accuracy in this dataset is higher than those in the former two as its fold change is larger than theirs. This finding is consistent with the conclusion proposed in [34]. For multivariate normal data with mean vectors *μ*_*1 *_and *μ*_*2 *_for the two classes, and common covariance matrix *Σ*, the optimal classifier has misclassification rate *exp{-(μ*_*1 *_*- μ*_*2*_*)' Σ*^*-1*^*(μ*_*1 *_*- μ*_*2*_*)}/2*. Thus, genes that are differentially expressed by a small amount are not particularly useful for classifying individual cases unless there are many uncorrelated such genes and the sample size is large enough to detect such genes without accepting numerous noise genes.

**Table 3:** Comparison of single gene classifiers and standard classifiers

Parameter Dataset	Smallest p-value^a^	t-test statistic^b^	Fold change^c^	*# *Significant gene^d^	Accuracy (%) of standard classifiers^e^	Accuracy (%) of single gene classifiers^f^
Melanoma	1.37e-29	22.68	277.78	7263	97	96.5

Breast Cancer 1	8.10e-06	9.06	3.65	20	52.2	66

Brain Cancer	1.51e-04	4.06	21.73	15	67	78.5

Breast Cancer 2	3.10e-06	5.16	3.48	180	70	54

Gastric Tumor	7.34e-10	9.51	10	4798	92.2	84.5

Lung Cancer 1	2.51e-21	20.34	1923.48	7561	97.2	96.5

Lung Cancer 2	6.82e-35	24.72	505.16	3219	99	93

Lymphoma	1.50e-04	4.07	1.33	2	58.5	72.5

Myeloma	5.00e-07	5.23	4.49	172	76.5	67.5

Pancreatic Cancer	1.30e-06	5.37	5.88	58	61	79.5

Prostate Cancer	1.34e-21	12.53	12.82	812	89.3	89

Note:

^a^The minimum univariate t-test p-value for the genes significantly different between the classes.

^b^The absolute value of the t-test statistic corresponding to the left smallest p-value.

^c^The maximum fold change in the geometry mean of gene expression between the classes,

^d^The total number of genes significantly different between the classes at 0.001 significance level.

^e^The mean classification accuracy of the four standard classifiers.

^f^The mean classification accuracy of the two single gene classifiers.

We also evaluated single gene classification on the datasets studied in [33]. In [33], the authors compared the classification results produced by some standard classifiers including those used in this study. They built classifiers based on selecting the 10, 50, 100, 500 and 1000 genes with the largest absolute t- and Wilcoxon statistics as well as all genes to conduct four classification experiments in two datasets [7,35]. Table S5 is a summary of results for those two datasets (Additional file 1).

We preprocessed the data as described in [33], and then performed a complete LOOCV to obtain the honest estimates of classification error. Table S6-9 list classification results for the single gene classifiers as well as part of the results presented in [33] for comparison (Additional file 1). These tables show that for the two datasets in [33], error rates for the single gene classifiers are generally close to those produced by standard methods. The one exception was the high error rate for the single gene classifier based on the Wilcoxon statistic in the Breast tumor estrogen dataset (see Table S6 of Additional file 1).

Two-gene classifiers have attracted a broad interest for their simplicity and interpretability, among which the top-scoring pair(s) (TSP) classifier was based on decision rules induced by comparing mRNA abundance in gene pairs [17]. We applied the TSP classifier to the eleven gene expression datasets and compared its performance to that of our single gene models (see Table 4). Table 4 demonstrates that the classification performance of our single gene classifiers is comparable to that of the TSP classifier. Our single gene classifiers have a substantial advantage over the TSP classifier in time efficiency for development and evaluation in cross validation.

**Table 4:** Comparison of classification accuracy (%) with the TSP classifier

Method Dataset	TSP	SGC-t	SGC-W
Melanoma	99	97	96

Breast Cancer 1	75	63	69

Brain Cancer	77	80	77

Breast Cancer 2	47	58	50

Gastric Tumor	91	89	80

Lung Cancer 1	95	98	95

Lung Cancer 2	97	93	93

Lymphoma	57	74	71

Myeloma	71	68	67

Pancreatic Cancer	90	69	90

Prostate Cancer	81	89	89

Note: The number of gene pairs selected is set as one for the TSP classifier.

The stability of the genes selected across the cross validation (CV) loop is also an important criterion to evaluate the usefulness of simple classifiers which involve a small number of genes. Table 5 presents all the genes selected and their occurrence percentages across the CV loop by the single gene classifiers in every dataset. Generally speaking, the genes selected across the CV loop with our methods are relatively stable (see Table 5).

**Table 5:** Stability of gene selection

Dataset	Classifier	The genes selected and their occurrence percentages across the CV loop
Melanoma	SGC-t	200965_s_at (99%), 213050_at (1%)
	
	SGC-W	217906_at (92%), 218552_at (4%), 218996_at (1%), 219343_at (1%), 221577_x_at (1%), 221882_s_at (1%)

Breast Cancer 1	SGC-t	259466 (92%), 291660 (5%), 950574 (3%)
	
	SGC-W	259466 (98%), 291660 (2%)

Brain Cancer	SGC-t	J02611_at (95%), X53331_at (5%)
	
	SGC-W	J02611_at (93%), X67951_at (3%), HG3543-HT3739_at (2%), X12794_at (2%),

Breast Cancer 2	SGC-t	AI868854 (65%), AK026899 (13%), AK026789 (12%), AK025709 (3%), AI240933 (3%), AF119844 (2%), AW006861 (2%)
	
	SGC-W	N30081 (65%), AF119844 (23%), AI868854 (12%)

Gastric Tumor	SGC-t	W70254 (100%)
	
	SGC-W	AA171606 (94%), W70254 (6%)

Lung Cancer 1	SGC-t	37210_at (66%), 198_g_at (15%), 40165_at (12%), 32254_at (5%), 41344_s_at (2%)
	
	SGC-W	1252_at (100%)

Lung Cancer 2	SGC-t	33754_at (100%)
	
	SGC-W	40936_at (98%), 33833_at (0.5%), 34320_at (0.5%), 37157_at (0.5%), 39640_at (0.5%)

Lymphoma	SGC-t	X76538_at (100%)
	
	SGC-W	X76538_at (91%), D30655_at (9%)

Myeloma	SGC-t	33146_at (88%), 32546_at (12%)
	
	SGC-W	32546_at (99%), 1071_at (1%)

Pancreatic Cancer	SGC-t	209596_at (98%), 206451_at (2%)
	
	SGC-W	206451_at (45%), 209596_at (43%), 218498_s_at (6%), 219625_s_at (4%), 212058_at (2%)

Prostate Cancer	SGC-t	34452_at (100%)
	
	SGC-W	34452_at (100%)

Note: In Breast Cancer 1, the genes are denoted by Clone ID; in Breast Cancer 2 and Gastric Tumor, the genes are denoted by GenBank Accession number; in all the others, the genes are denoted by Probe Set.

## Discussion

In contrast to most of the data investigated in traditional machine learning and data mining applications which are often composed of low-dimensional attributes and high-dimensional instances, microarray data are composed of high-dimensional attributes (p) and low-dimensional instances (n). Consequently some traditional machine learning and data mining algorithms which are effective for the former become ineffective for some p > n problems like microarray classification. Excellent classification can in some cases be achieved with a small number of genes, even a single gene selected from thousands of candidates. Optimal complexity depends on the degree of differential expression among the classes and sample size. Complexity is not, however simply the number of genes in the classifiers. Complexity also depends on gene selection criteria and classification rules employed. Simple models typically involve a simple feature selection scheme and simple classification rule. In contrast, complex models often involve sophisticated feature selection procedures and/or complicated classification rules. Models based on complex algorithms for multivariate gene selection and complex classification rules may contain few genes but overfit the data. Empirical comparisons have indicated that complicated wrapper methods such as aggregated classification trees sometimes perform poorly compared to simple classifiers such as DLDA and *k*-NN in some cases [36].

Gene selection is critical in building good classifiers and there is no simple completely general answer to the question of how many genes a good classifier should include? For interpretability and ease of porting to assay platforms more suitable to use in clinical practice, it is advantageous to include a small number of genes in the classifier. The optimal number of genes depends on the sample size, the number of differentially expressed genes, their degree of differential expression and correlation structure and the type of classifier used [34,37,38]. In some cases, the number of genes or other aspects of classifier complexity can be regarded as tuning parameters to be optimized by an inner-loop of cross-validation [39]. Our results indicate that single-gene models should be included as candidate classifiers in such optimization.

In [33], the authors explored the sensitivity to number of features for some standard classifiers, and found only limited changes in performance when varying the number of genes used with a lower limit of 10 genes. Classification accuracy with 10 genes was in most cases as good as or better than accuracy with more genes. Although the univariate feature selection approach used by Dudoit and Fridyland was simple compared to some of the complex multivariate feature selection approaches that have been used, the former often outperformed the latter [29,40].

We have found that single gene classification models are frequently of commensurate accuracy as more complex classifiers. For problems with genes that are quite differentially expressed, single gene classifiers appear to do well. For more difficult problems without highly differentially expressed genes, it can be useful to include more genes in the model instead of using the single most extreme gene which may be noise. In some of these cases with small number of samples, however, the single gene model might do as well because models with more genes may overfit the data.

For most of the datasets examined, the single-gene classification methods appear to work as well as more standard methods such as DLDA, SVM, *k*-NN and RF, based on a larger number of genes, and two-gene classifiers such as the TSP classifier. Here the classification results used for comparison obtained by DLDA, SVM, *k*-NN and RF might not be optimal as we have pre-specified their model parameters rather than optimized these parameters. Thus, we re-examined the classification results obtained through optimizing the parameters of the compared classification models. For *k*-NN, we compared the three groups of classification results obtained by1-NN, 3-NN and the nearest centroid, respectively. These results were close to each other (see Table S10 of Additional file 1). Furthermore, we re-classified the eleven data sets using the DLDA, *k*-NN and SVM classifiers with the optimized gene selection significance level which was chosen from the grid 0.01, 0.005, 0.001, and 0.0005 in order to minimize the CV error rate. Table S11 presents the classification accuracies attained with the optimized and no- optimized parameter for all of the datasets (Additional file 1), suggesting that their gap is minor. In addition, we examined the classification results achieved under varied values of tuning parameter cost for SVM for selected datasets and found no change. Finally, we investigated the performance variation of the RF classifier by tuning its two parameters: the number of trees and the number of genes randomly sampled as candidates at each split. We found that the performance variation was minimal. In summary, the classification performance with the pre-specified parameters is close to that with the optimized parameters for DLDA, SVM, *k*-NN and RF, and therefore the conclusions gained from the comparison analyses of our single-gene classifiers and the standard classifiers are justified.

Our single gene classifier for a training set was developed by applying the entropy-based discretization method to find the optimal cut point for the single gene selected based on the t or WMW statistics (see the Methods). Of course, the cut-point finding could also be included in the single gene selection like the methods proposed in [20,41]. However, our experiments have indicated that the cut-point based feature selection for all genes would greatly compromise the time efficiency of the algorithm for high-dimensional gene expression data which generally contain thousands of attributes. Additionally, the gene selection methods involved in discretization may miss the most informative genes because the discretization procedure itself could cause the partial loss of the information hidden behind data. By contrast, the t-test or WMW based gene selection approach can avoid of this kind of information loss, and therefore is more likely to select the most informative genes. Including optimal discretization in gene selection could also result in overfitting the training set. This issue could be addressed in future research.

## Conclusions

To deal with high-dimensional gene expression data, simple classifiers should be preferred to complicated ones for their interpretability and applicability. In the present study, we developed extremely simple single-gene classifiers. We examined a large number of datasets and a large number of previously published classifier algorithms and found that our single gene classifiers have comparable performance to more complex classifiers in most cases examined. Our algorithm for development of single gene classifiers is computationally efficient and the single gene developed appears reasonably stable. Although single gene classifiers are not always successful, their examination is worthwhile because of their advantages for interpretability and applicability for biological study and medical use.

## Methods

### Classifier Development

Within each training set, we used the t-test or the Wilcoxon-Mann-Whitney (WMW) test to identify the most statistically significant gene(s) in distinguishing the two classes. If there were multiple genes with the smallest p-value (very rare), we chose the one with the smallest order number in the dataset. Although the t-test is a popularly used feature selection method, it is sensitive to gross errors in the data [42]. Alternatively, the WMW test is a rank-based test which is robust to errors in the data. Therefore we evaluated it for gene selection as an alternative to the t-test.

Once the single gene was selected for the training set, we constructed the classification rule based on a single cut-point for the expression levels of that gene. If the expression level of gene *g *in the sample *s *is no more than *T*, then the sample is assigned to the class *c*_*1*_; otherwise the sample is assigned to the class *c*_*2*_, i.e., "*E(g, s) *≤ *T = > C(s) = c*_*1*_; *E(g, s) > T = > C(s) = c*_*2*_". Here we refer to the *T *as the optimal cut point for gene *g*. We found the optimal cut point by using the entropy-based discretization method [43].

For each training set *S*, we determined the gene *g *with the most significant t or WMW statistic, then sorted the samples as *s*_*1*_*, s*_*2*_*, ..., s*_*n*_, based on the expression levels of the selected gene *g*. We constructed the candidate cut point set *P *which was composed of the mean values of *E(g, s*_*k*_*) *and *E(g, s*_*k+1*_*) *for all *C(s*_*k*_*)*≠*C(s*_*k+1*_*)*. For each candidate cut point *t *∈ *P*, we partitioned *S *into two equivalence classes *S*_*1 *_and *S*_*2*_, where *S*_*1 *_*= *{*s *∈ *S *| *E(g, s) *≤ *t*} *and S*_*2 *_*= *{*s *∈ *S *| *E(g, s) > t*}. Let *C*_*1 *_denote the subset of samples whose class label is *c*_*1*_, and *C*_*2 *_the subset of samples whose class label is *c*_*2*_. Define the four sets: *P*_*11*_, *P*_*12*_, *P*_*21 *_and *P*_*22*_, where *P*_*11*_*= S*_*1 *_∩ *C*_*1*_, *P*_*12*_*= S*_*1 *_∩ *C*_*2*_, *P*_*21*_*= S*_*2 *_∩ *C*_*1*_, and *P*_*22*_*= S*_*2 *_∩ *C*_*2*_. We calculated the class information entropy of the partition induced by *t*, denoted *E(g, t, S)*, which is given by:

$$E\left(g,t,S\right)=-\frac{\left|S_{1}\right|}{\left|S\right|}\left(\frac{\left|P_{11}\right|}{\left|S_{1}\right|}log_{2}\frac{\left|P_{11}\right|}{\left|S_{1}\right|}+\frac{\left|P_{12}\right|}{\left|S_{1}\right|}log_{2}\frac{\left|P_{12}\right|}{\left|S_{1}\right|}\right)-\frac{\left|S_{2}\right|}{\left|S\right|}(\frac{\left|P_{21}\right|}{\left|S_{2}\right|}log_{2}\frac{\left|P_{21}\right|}{\left|S_{2}\right|}+\frac{\left|P_{22}\right|}{\left|S_{2}\right|}log_{2}\frac{\left|P_{22}\right|}{\left|S_{2}\right|}).$$

We selected the *t *which minimized *E(g, t, S) *as the optimal cut point *T *produced by the gene *g*. If the candidate cut point set *P *was empty (very rare), we took the mean expression level of gene *g *in all training samples as the optimal cut point.

Here |*P*_*11*_| and |*P*_*22*_| denote the number of class 1 and class 2 samples predicted correctly respectively, and |*P*_*21*_| and |*P*_*12*_| denote the number of class 1 and class 2 samples predicted incorrectly. We adopt the classification rule "*E(g, s) *≤ *T = > C(s) = c*_*1*_; *E(g, s) > T = > C(s) = c*_*2*_" if |*P*_*11*_| + |*P*_*22*_| > |*P*_*12*_| + |*P*_*21*_|. However, if |*P*_*11*_| + |*P*_*22*_| ≤ |*P*_*12*_| + |*P*_*21*_|, then we reverse the direction of classification, i.e., "*E(g, s) *≤ *T = > C(s) = c*_*2*_; *E(g, s) > T = > C(s) = c*_*1*_".

### Measuring Classifier Performance

We used complete leave-one-out cross validation (LOOCV) to evaluate classifier performance. All components of classifier development were repeated within each loop of the cross-validation; i.e. in each leave-one-out training set we selected a single gene and a single cut-point for that gene and used that single classifier to classify the omitted sample. In addition, we also conducted the validation by randomly separating the samples into one training set and one test set. For each data set, we carried out two types of separations: Type 1 separation (the sample size in the training set is approximately equal to that in the test set), and Type 2 separation (the sample size in the training set is about twice as that in the test set) (see Table S12 and Table S13 of Additional file 1). Thus, we obtained three sets of classification accuracy results. The LOOCV results are presented in this text, while the other two are presented in Additional file 1.

### Materials

We selected eleven gene expression datasets to evaluate classifier performance. These datasets were selected to cover the range of sample size, gene number and degree of classification difficulty. The Lung Cancer 2 and Myeloma datasets have large sample size. The Melanoma, Brain Cancer, Breast Cancer 2, Lung Cancer 1, Lymphoma and Pancreatic Cancer have relatively small sample size. The sample size of the Breast Cancer 1, Gastric Tumor and Prostate Cancer is intermediate. The Melanoma, Breast Cancer 2, Gastric Tumor and Pancreatic Cancer datasets contain a large number of genes, the Breast Cancer 1, Brain Cancer and Lymphoma datasets involve a relatively small number of genes and the gene numbers in the other datasets are intermediate. As for the degree of classification difficulty, the Melanoma, Gastric Tumor, Lung Cancer 1, Lung Cancer 2 and Prostate Cancer are easily-classified datasets, while the others are more difficult. The datasets are described in Table 6.

**Table 6:** Summary of the eleven gene expression datasets

Dataset	# Genes	Class	# Samples
Melanoma [45]	22283	malignant/nonmalignant	70 (45/25)

Breast Cancer 1 [46]	7650	relapse/no-relapse	99 (45/54)

Brain Cancer [7]	7129	classic/desmoplastic	60 (46/14)

Breast Cancer 2 [47]	22575	disease-free/cancer recurred	60 (32/28)

Gastric Tumor [48]	19508	normal/tumor	132 (29/103)

Lung Cancer 1 [49]	12600	squamous cell lung carcinoma/pulmonary carcinoid	41 (21/20)

Lung Cancer 2 [3]	12533	mesothelioma/adenocarcinoma	181 (31/150)

Lymphoma [8]	7129	cured/fatal	58 (32/26)

Myeloma [50]	12651	without bone lytic lesion/with bone lytic lesion	173 (36/137)

Pancreatic Cancer [51]	22283	normal/pancreatic ductal carcinoma	49 (25/24)

Prostate Cancer [6]	12600	normal/tumor	102 (50/52)

Note: The sample size of each class is given in parenthesis.

### Standard Classification Methods Used for Comparison

We compared the performance of our models to that of four standard classifiers: DLDA, *k*-NN, RF and SVM. For the *k*-NN classifier, we set the parameter *k *as 3. For the RF classifier, we set the number of trees and genes randomly sampled as candidates at each split as 100 and the squared root of the total number of genes, respectively. The SVM is based on the linear inner product kernel function.

For the four classifiers, the genes significantly different between the classes at 0.001 significance level were used for class prediction. We carried out the four classification algorithms in BRB-ArrayTools, which is an integrated package for the visualization and statistical analysis of DNA microarray gene expression data [44]. The software can be freely downloaded from the website: http://linus.nci.nih.gov/BRB-ArrayTools.html.

## Authors' contributions

XW and RS designed the research. XW and RS wrote the manuscript. XW performed the data analyses and programming for the plugin for BRB-ArrayTools. Both authors read and approved the final manuscript.

## Supplementary Material

Additional file 1 **Supplementary Table S1-13**. The list of 13 supplementary tables Click here for file
