# Supplementary material for: Microarray-based cancer prediction using single genes
Source: BMC Bioinformatics. 2011 Oct 7;12:391. doi: 10.1186/1471-2105-12-391 (PMC3228540; doi:10.1186/1471-2105-12-391)
Supplement: Additional file 1 — Supplementary Table S1-13. The list of 13 supplementary tables [file 1471-2105-12-391-S1.DOC]

**Table S1. The classification accuracy (%) in the case of Type 1 separation**

| Method SGC-t SGC-W DLDA *k*-NN SVM RF  Dataset |
| --- |
| Melanoma 91 85 96 100 96 100 |
| Breast Cancer 1 30 61 33 33 30 30 |
| Brain Cancer 65 65 70 75 50 65 |
| Breast Cancer 2 55 45 65 75 70 70 |
| Gastric Tumor 87 61 91 89 91 91 |
| Lung Cancer 1 100 100 96 96 96 100 |
| Lung Cancer 2 95 98 98 100 100 100 |
| Lymphoma 55 55 55 45 55 55 |
| Myeloma 60 60 72 78 79 81 |
| Pancreatic Cancer 88 75 75 69 81 56 |
| Prostate Cancer 82 82 82 85 85 88 |

**Table S2. The classification accuracy (%) in the case of Type 2 separation**

| Method SGC-t SGC-W DLDA *k*-NN SVM RF  Dataset |
| --- |
| Melanoma 91 94 97 100 100 100 |
| Breast Cancer 1 41 53 24 35 22 27 |
| Brain Cancer 57 60 60 70 70 67 |
| Breast Cancer 2 60 67 50 57 53 57 |
| Gastric Tumor 88 82 76 83 83 83 |
| Lung Cancer 1 100 85 95 100 100 95 |
| Lung Cancer 2 88 97 100 100 99 96 |
| Lymphoma 28 59 28 45 34 38 |
| Myeloma 55 33 30 55 40 43 |
| Pancreatic Cancer 24 52 32 32 28 20 |
| Prostate Cancer 41 80 31 43 47 41 |
|  |

**Table S3. The number of genes in classifiers in the case of Type 1 separation**

| Method SGC-t SGC-W DLDA *k*-NN SVM RF  Dataset |
| --- |
| Melanoma1 1 1 5441 5441 5441 2083 |
| Breast Cancer 1 1 1 61 61 61 57 |
| Brain Cancer 1 1 33 33 33 33 |
| Breast Cancer 2 1 1 118 118 118 118 |
| Gastric Tumor 1 1 2701 2701 2701 531 |
| Lung Cancer 1 1 1 5124 5124 5124 1450 |
| Lung Cancer 2 1 1 2057 2057 2057 2057 |
| Lymphoma1 1 1 173 173 173 162 |
| Myeloma 1 1 119 119 119 64 |
| Pancreatic Cancer 1 1 14 14 14 9 |
| Prostate Cancer 1 1 579 579 579 579 |

**Table S4. The number of genes in classifiers in the case of Type 2 separation**

| Method SGC-t SGC-W DLDA *k*-NN SVM RF  Dataset |
| --- |
| Melanoma 1 1 4948 4948 4948 1878 |
| Breast Cancer 1 1 1 260 260 260 261 |
| Brain Cancer 1 1 4 4 4 4 |
| Breast Cancer 2 1 1 30 30 30 30 |
| Gastric Tumor 1 1 1695 1695 1695 1715 |
| Lung Cancer 1 1 1 4703 4703 4703 1375 |
| Lung Cancer 2 1 1 1511 1511 1511 1156 |
| Lymphoma 1 1 975 975 975 92 |
| Myeloma 1 1 586 586 586 204 |
| Pancreatic Cancer 1 1 557 557 557 478 |
| Prostate Cancer 1 1 4374 4374 4374 3858 |

**Table S5. Summary of the two gene expression datasets used in ref. 33**

| Dataset # Genes Class # Samples | |
| --- | --- |
| Breast Cancer 7129 | ER+ / ER- ^g^ 49 (25 /24) |
|  | node + / node- ^h^ 49 (25 /24) |
| Brain Cancer 7129 | desmoplastic / classic ^i^ 34 (9 /25) |
|  | survivor / nonsurvivor ^j^ 60 (39 /21) |

Note:

^g^ Estrogen receptor status, ER+ (25 samples) vs. ER- (24 samples).

^h^ lymph node status, affected node present or node+ (25 samples) vs. affected node absent or node- (24 samples).

^i^ 34 medulloblastoma samples, 9 of which are desmoplastic and 25 classic.

^j^ 60 medulloblastoma samples, corresponding to 39 survivors and 21 nonsurvivors.

**Table S6. Classification error in Breast tumor estrogen dataset**

| # Gene in classifier 1 10 50 100 500 1000 7129  Classifier | |
| --- | --- |
| DLDA | t-stat 6 3 4 4 4 6 |
|  | W-stat 6 4 4 4 4 6 |
| *k*-NN | t-stat 5 5 4 7 5 6 |
|  | W-stat 5 5 4 6 5 6 |
| SVM | t-stat 5 4 4 7 6 6 |
|  | W-stat 7 3 3 7 6 6 |
| RF | t-stat 3 3 5 5 5 4 |
|  | W-stat 2 3 4 5 5 4 |
| DQDA | t-stat 5 4 6 6 7 15 |
|  | W-stat 6 4 4 6 7 15 |
| LogitBoost | t-stat 4 1 2 3 5 3 |
|  | W-stat 4 3 2 3 4 3 |
| Our method | t-stat 4 |
|  | W-stat 19 |

Note: *k*-NN is 3-NN; SVM is linear with *C*=100; RF is with the genes randomly sampled as candidates at each split equal to the squared root of the total number of genes used; LogitBoost is with *B*=100 (Dudoit and Fridlyand 2003). The same parameters apply to Table S7-9.

**Table S7. Classification error in Breast tumor nodal dataset**

| # Gene in classifier 1 10 50 100 500 1000 7129  Classifier | |
| --- | --- |
| DLDA | t-stat 8 14 19 17 15 17 |
|  | W-stat 10 10 15 17 16 17 |
| *k*-NN | t-stat 8 22 19 19 22 26 |
|  | W-stat 10 16 18 19 21 26 |
| SVM | t-stat 9 14 21 12 15 20 |
|  | W-stat 11 18 16 14 14 20 |
| RF | t-stat 7 13 14 15 17 18 |
|  | W-stat 8 9 15 17 17 18 |
| DQDA | t-stat 11 17 18 22 24 26 |
|  | W-stat 12 13 22 22 20 26 |
| LogitBoost | t-stat 9 21 18 14 14 12 |
|  | W-stat 9 13 21 11 11 12 |
| Our method | t-stat 13 |
|  | W-stat 24 |

**Table S8. Classification error in Brain tumor classic vs. desmoplastic**

| # Gene in classifier 1 10 50 100 500 1000 7129  Classifier | |
| --- | --- |
| DLDA | t-stat 6 4 5 4 4 5 |
|  | W-stat 4 5 5 4 4 5 |
| *k*-NN | t-stat 5 5 3 4 5 8 |
|  | W-stat 4 4 3 4 4 8 |
| SVM | t-stat 4 8 7 7 6 6 |
|  | W-stat 9 7 7 6 5 6 |
| RF | t-stat 6 6 6 8 9 9 |
|  | W-stat 5 7 7 9 9 9 |
| DQDA | t-stat 6 6 6 8 8 9 |
|  | W-stat 5 6 5 5 9 9 |
| LogitBoost | t-stat 12 7 8 7 7 9 |
|  | W-stat 6 5 7 8 9 9 |
| Our method | t-stat 9 |
|  | W-stat 6 |

**Table S9. Classification error in Brain tumor MD survival dataset**

| # Gene in classifier 1 10 50 100 500 1000 7129  Classifier | |
| --- | --- |
| DLDA | t-stat 19 25 23 24 24 26 |
|  | W-stat 17 26 21 24 25 26 |
| *k*-NN | t-stat 20 24 22 20 19 23 |
|  | W-stat 16 22 19 18 21 23 |
| SVM | t-stat 19 29 29 22 22 19 |
|  | W-stat 21 28 25 24 24 19 |
| RF | t-stat 18 21 20 19 20 22 |
|  | W-stat 17 20 18 19 20 22 |
| DQDA | t-stat 22 24 23 25 23 23 |
|  | W-stat 17 27 22 27 26 23 |
| LogitBoost | t-stat 19 19 26 16 16 16 |
|  | W-stat 21 24 26 19 15 16 |
| Our method | t-stat 22 |
|  | W-stat 17 |

**Table S10. Comparison of *k*-NN classification accuracy (%) for different *k*** value

| Method 1-NN 3-NN Nearest Centroid  Dataset |
| --- |
| Melanoma 99 97 94 |
| Breast Cancer 1 52 53 62 |
| Brain Cancer 65 73 62 |
| Breast Cancer 2 72 67 72 |
| Gastric Tumor 98 96 80 |
| Lung Cancer 1 98 98 95 |
| Lung Cancer 2 100 99 99 |
| Lymphoma 55 52 55 |
| Myeloma 76 78 79 |
| Pancreatic Cancer 73 61 65 |
| Prostate Cancer 88 93 76 |

**Table S11. Classification accuracy (%) at the optimized gene selection significance level**

| Method DLDA *k*-NN (*k*=3) SVM  Dataset |
| --- |
| Melanoma 97 (97) 97 (97) 97 (97) |
| Breast Cancer 1 60 (61) 49 (53) 55 (52) |
| Brain Cancer 68 (65) 72 (73) 73 (60) |
| Breast Cancer 2 72 (73) 68 (67) 67 (73) |
| Gastric Tumor 85 (81) 95 (96) 97 (97) |
| Lung Cancer 1 95 (95) 98 (98) 98 (98) |
| Lung Cancer 2 99 (99) 99 (99) 99 (99) |
| Lymphoma 69 (66) 59 (52) 55 (59) |
| Myeloma 75 (75) 76 (78) 72 (74) |
| Pancreatic Cancer 59 (63) 61 (61) 63 (65) |
| Prostate Cancer 78 (78) 92 (93) 92 (93) |

Note:

1 The classification accuracy was obtained with the optimized gene selection significance level chosen from the grid 0.01, 0.005, 0.001, and 0.0005 to minimize the CV error rate. In our case, the gene selection significance level was specified as 0.001.

2 The numbers out of parenthesis are the optimal classification results while the numbers in parenthesis are the results obtained at the 0.001significance level.

**Table S12. Summary of the Type 1 separation of datasets**

**(# training sample: # test sa**mple≈2:1)

| Dataset Class # Training samples # Test samples |
| --- |
| Melanoma malignant / no-malignant 47 (30 / 17) 23 (15 / 8) |
| Breast Cancer 1 relapse / no-relapse 66 (30 / 36) 33 (15 / 18) |
| Brain Cancer Classic / Desmoplastic 40 (31 / 9) 20 (15 / 5) |
| Breast Cancer 2 disease-free / cancer recurred 40 (21 / 19) 20 (11 / 9) |
| Gastric Tumor normal / tumor 88 (19 / 69) 44 (10 / 34) |
| Lung Cancer 1 squamous cell lung carcinoma 27 ( 14 / 13) 14 (7 / 7)  / pulmonary carcinoid |
| Lung Cancer 2 mesothelioma / adenocarcinoma 121 (21 / 100) 60 (10 / 50) |
| Lymphoma cured / fatal 38 (21 / 17) 20 (11 / 9) |
| Myeloma without bone lytic lesion 115 (24 / 137) 58 (12 / 137)  / with bone lytic lesion |
| Pancreatic Cancer normal / pancreatic ductal carcinoma 33 (17 / 16) 16 (8 / 8) |
| Prostate Cancer normal / tumor 68 (33 / 35) 34 (17 / 17) |

**Table S13. Summary of the Type 2 separation of datasets**

(# training sample: # test sample≈1:1)

| Dataset Class # Training samples # Test samples |
| --- |
| Melanoma malignant / no-malignant 35 (23 / 12) 35 (22 / 13) |
| Breast Cancer 1 relapse / no-relapse 50 (27 / 23) 49 (27 / 22) |
| Brain Cancer Classic / Desmoplastic 30 (23 / 7) 30 (23 / 7) |
| Breast Cancer 2 disease-free / cancer recurred 30 (16 / 14) 30 (16 / 14) |
| Gastric Tumor normal / tumor 66 (15 / 51) 66 (14 / 52) |
| Lung Cancer 1 squamous cell lung carcinoma 21 (11 / 10) 20 (10 / 10)  / pulmonary carcinoid |
| Lung Cancer 2 mesothelioma / adenocarcinoma 91 (16 / 75) 90 (15 / 75) |
| Lymphoma cured / fatal 29 (16 / 13) 29 (16 / 13) |
| Myeloma without bone lytic lesion 87 (18 / 69) 86 (18 / 68)  / with bone lytic lesion |
| Pancreatic Cancer normal / pancreatic ductal carcinoma 24 (12 / 12) 25 (13 / 12) |
| Prostate Cancer normal / tumor 51 (25 / 26) 51 (25 / 26) |
